# *Staphylococcus aureus* in Horses in Nigeria: Occurrence, Antimicrobial, Methicillin and Heavy Metal Resistance and Virulence Potentials

**DOI:** 10.3390/antibiotics12020242

**Published:** 2023-01-24

**Authors:** Obichukwu Chisom Nwobi, Madubuike Umunna Anyanwu, Ishmael Festus Jaja, Innocent Okwundu Nwankwo, Chukwuemeka Calistus Okolo, Chibundo Adaobi Nwobi, Ekene Vivienne Ezenduka, James Wabwire Oguttu

**Affiliations:** 1 Department of Veterinary Public Health and Preventive Medicine, University of Nigeria, Nsukka 400001, Nigeria; 2 Microbiology Unit, Department of Veterinary Pathology and Microbiology, University of Nigeria, Nsukka 400001, Nigeria; 3 Department of Agriculture and Animal Health, Florida Campus, University of South Africa, Johannesburg 1709, South Africa; 4 Department of Veterinary Medicine, University of Nigeria, Nsukka 400001, Nigeria; 5 Department of Home Science and Management, University of Nigeria, Nsukka 400001, Nigeria

**Keywords:** equine, heavy metal tolerance, methicillin resistance, *Staphylococcus aureus*, virulence

## Abstract

*Staphylococcus aureus* was isolated from a total of 360 nasal and groin skin swabs from 180 systematic randomly-selected horses slaughtered for meat at Obollo-Afor, Enugu State, Southeast Nigeria and antimicrobial, methicillin and heavy metal resistance profile and virulence potentials of the isolates established. Baird-Parker agar with egg yolk tellurite was used for *S*. *aureus* isolation. *S*. *aureus* isolates were confirmed biochemically and serologically using a specific *S*. *aureus* Staphytect Plus™ latex agglutination test kit. The antimicrobial resistance profile, methicillin, vancomycin and inducible clindamycin resistance, and β-lactamase production of the isolates were determined with disc diffusion. Tolerance to Copper, Cadmium, Lead and Zinc was assessed using the agar dilution method and virulence potentials were determined using phenotypic methods. Forty-three (23.9%) of the 180 horses harbored *S*. *aureus*. Some 71 *S*. *aureus* were recovered from the 360 samples. Two (2.8%) of the 71 *S*. *aureus* were methicillin-resistant *S*. *aureus* (MRSA) and 69 (97.2%) were methicillin-susceptible. MRSA was recovered from 2 (1.1%) of the 180 horses. Some 9.4% of the isolates were multiple drug-resistant (MDR). The mean multiple antibiotic resistance indices (MARI) for the isolates was 0.24. Heavy metal resistance rate of the isolates ranged between 35.4–70.4%. The isolates, including the MRSA strains, displayed virulence potentials as clumping factor and catalase, gelatinase, caseinase, heamolysin, and biofilm was at the rate of 100%, 53.5%, 43.7%, 18.3% and 23.9%, respectively. This study showed that a considerable percentage of horses slaughtered in Obollo-Afor Southeastern Nigeria are potential reservoirs of virulent multiple drug- and heavy metal-resistant *S*. *aureus*, including MRSA, that could spread to humans and the environment.

## 1. Introduction

*Staphylococcus* (*S*.) *aureus* is concurrently a zoonotic commensal and pathogen incriminated in several life-threatening diseases ranging from cosmetic to lethal manifestations in humans and animals, including horses [1,2]. In 2019 alone, *S*. *aureus*, both antimicrobial-resistant and non-resistant strains was the leading cause of human death and responsible for the death of up to 1 million people [3,4]. Thus, as the World Health Organization recommended, there is a need for increased surveillance of *S*. *aureus* in various reservoirs [5]. The shedding of *S*. *aureus* from mucous membranes and skin by colonized individuals results in contamination of the immediate environment, from where this organism is transmitted to other ecological niches [6]. The population of horses in Nigeria is estimated at 200,000–240,000 [7]. Horses are used for various purposes (including for sports-polo games, security-mountain troops, festivals-dubar, etc.) in the northern region of Nigeria and are often transported to the southeastern region of the federation where they are slaughtered for meat [7]. These horses are slaughtered and processed in an unhygienic environment, often laden with microorganisms, including staphylococci, by inadequately trained personnel with poor personal hygiene/infection prevention and control practices [8]. Therefore, horse meats processed in Nigeria may become contaminated when encountering contaminated slaughterhouse environment and equipment, colonized slaughterhouse personnel, and/or polluted water used for washing the horse carcass [9]. 

Occupational contact with animals and meat products is a risk factor for the acquisition of *S*. *aureus* from animals [1,9]. It is established that *S*. *aureus* colonizes horses and their handlers [10,11,12,13,14]. Slaughterhouse personnel (such as butchers, meat carcass processors and cleaners), and consumers/handlers of horse meat are more at risk of acquiring *S*. *aureus* from infected/colonized horses since these groups frequently contact the horses and their carcasses [9,14,15]. When colonized by *S*. *aureus*, these individuals subsequently transfer the infection to their households or communities. 

Inappropriate use of antimicrobial agents has led to the development of resistant staphylococci, including methicillin-resistant *S*. *aureus* (MRSA) strains. The use of antimicrobial agents as prophylactics and therapy without conducting sensitivity tests for fatal equine diseases such as *Rhodococcus equi* infection, sepsis and endotoxaemia is allowed globally [16,17,18,19]. Moreover, in Nigeria, antimicrobial agents, including the critically-important ones, are used by non-professionals in the management of animals, including horses, without the supervision of a veterinarian [7,20]. Thus, horses in Nigeria might be exposed to various antimicrobial agents and could as well be acquiring *S*. *aureus* from anthropogenic- and agricultural-impacted environments. Antimicrobial-resistant *S*. *aureus*, especially MRSA, is of food safety concern since the superbug poses a public health threat by being refractory to major antimicrobial classes in routine use and is a consistent etiology of outbreaks [21]. Moreover, MRSA infections are synchronous with extended hospital stays coupled with increased healthcare costs [22,23,24]. Furthermore, the carriage by animals and humans of *S*. *aureus* (both methicillin-susceptible *S*. *aureus* [MSSA] and MRSA) resistant to critically-important last-resort drugs such as clindamycin, vancomycin and macrolides used in treating infections associated with MRSA is of serious public health concern since infections by such organisms result in high morbidity, hospital cost, and mortality [25,26]. Bacterial co-expression of antibiotic and heavy metal resistance, especially by *S*. *aureus* is an emerging global public health issue, as resistance to one confers resistance to the other [27,28,29]. This co-resistance results in the development of organism which are refractory to both antibiotics and disinfectants. Such organisms can jeopardize antimicrobial therapy in colonized/infected individuals and can breach biosecurity measures in farms, hospitals, and households [27]. Asides from resistance to antimicrobials and heavy metals, the expression of virulence factors (VFs) makes *S*. *aureus* one of the most important pathogens of public health concern. Among the VFs expressed by *S*. *aureus*, catalase, clumping factor (bound coagulase), haemolysin, biofilm, gelatinase, caseinase, amylase, and lipase helps in host invasion and colonization [30,31,32,33]. Assessing antimicrobial/heavy metal susceptibility and virulence potentials of *S*. *aureus* from horses is crucial for targeted empirical therapy and tailored prevention and control measures in the equine industry/horse meat chain to help reduce *S*. *aureus*-associated morbidity and mortality in humans and animals.

Studies on the occurrence, antimicrobial susceptibility, and virulence of equine *S*. *aureus* have been done in Australia [34,35], and countries in Asia [36,37,38,39], North America [40] and Europe [10,41,42,43]. In Nigeria, there is a paucity of information about *S*. *aureus* in horses [44]. The occurrence, antimicrobial and heavy metal susceptibility, and virulence potentials of *S*. *aureus* in horses slaughtered in Nigeria remain uninvestigated. Monitoring resistant and pathogenic organisms in horses slaughtered in Nigeria is critical to devise mitigation strategies and empiric treatment of infections associated with the organisms in the study area. Therefore, this study has aimed to isolate *S*. *aureus* from ready-to-slaughter horses in Obollo-Afor Southeast Nigeria, detect methicillin-resistant *S*. *aureus* strains, establish the antimicrobial and heavy metal resistance profile, and elucidate virulence potentials of the isolates. 

## 2. Materials and Methods

### 2.1. Study Area

The study was conducted at Obollo-Afor horse slaughter slab in Udenu Local Government Area, Enugu State Southeast Nigeria. Obollo-Afor is geographically located at coordinates 6.9153° N and 7.5139° E. Obollo-Afor market is a major horse-selling and slaughtering point in Southeast Nigeria. Horses constitute a major source of animal protein to the Udenu populace. An average of 15 horses are slaughtered daily with approximately 200 horses slaughtered per month.

### 2.2. Sample Collection, Bacterial Isolation and Identification

One hundred and eighty horses which made up about 15% of the total slaughter between February and June 2019, were selected using systematic one-in-five random sampling techniques. Before slaughter, a single nasal and skin (groin) swab was collected from each horse. The cotton-tipped swabs were inserted approximately 10 cm into one nasal passage and withdrawn while contacting the nasal mucosa with the swab. Without prior cleaning or disinfection, swabs whose cotton tips were moistened with sterile normal saline were used in swabbing about 5 cm^2^ of the groin skin area. The nasal and skin swab samples were transported with ice packs to the Laboratory of the Department of Veterinary Public Health and Preventive Medicine, University of Nigeria, and processed immediately upon arrival for *S*. *aureus* isolation. Each swab was inoculated into 5 mL of nutrient broth containing 6.5% NaCl and incubated at 37 °C for 24 h. A loopful of the broth culture was streaked on Baird-Parker agar (BPA) containing egg yolk tellurite (EYT) and incubated at 37 °C for 24–48 h. Different morphological types were observed and described accordingly. Suspected *S*. *aureus* colonies (shiny black colonies with clear halos with or without opaque zones) were picked from each plate and purified by inoculating on fresh BPA with EYT and incubated at 37 °C for 24 h. Colonies of pure cultures of the isolates were further sub-cultured on Mannitol salt agar (MSA), and incubated at 37 °C for 24 h. Mannitol-fermenting (yellow) colonies on MSA were morphologically and biochemically confirmed as *Staphylococcus* using the Gram staining and catalase test, respectively. The *S*. *aureus* isolates were confirmed serologically using the *S*. *aureus* Staphytect Plus™ latex agglutination identification kit (Oxoid, Hampshire England) (which detects clumping factor, Protein A and capsular polysaccharides) according to the manufacturer’s instructions. *S*. *aureus* ATCC 25923 and *S*. *epidermidis* ATCC 12228 strains were used as a positive and negative control, respectively. Isolates confirmed as *S*. *aureus* strains were sub-cultured onto nutrient agar slants, incubated at 37 °C for 24 h, and stored in the refrigerator for a maximum of 48 h at 4 °C as stock cultures until further analysis [9]. 

### 2.3. Antimicrobial Resistance Profile

Antimicrobial susceptibility testing of the *S*. *aureus* isolates was performed with the disc diffusion method according to the guidelines of the Clinical Laboratory Standards Institute [45] with discs (Oxoid Hampshire England) impregnated with the following 11 antimicrobial agents belonging to 10 classes: β-lactam–penicillin (PEN, 10 units), cefoxitin (CFT, 30 µg), aminoglycosides–gentamicin (GEN, 10 µg), oxazolidinones–linezolid (LZD, 30 µg), lincosamides-clindamycin (CLI, 2 µg), ansamycins–rifampicin (RIF, 5 µg), tetracycline (TET, 30 µg), macrolides–erythromycin (ERY, 15 µg), glycopeptides–vancomycin (VAN, 30 µg), fluoroquinolones–ciprofloxacin (CIP, 5 µg) and folate pathway inhibitor–trimethoprim-sulphamethoxazole (1.25/23.75 µg). *S*. *aureus* ATCC 25923 was used as a quality-control strain for susceptibility. Inhibition zone diameters were interpreted in accordance with the breakpoints for *Staphylococcus* [45]. Multiple antimicrobial resistance index (MARI) of each isolate was established using the formula a/b where ‘a’ is the number of antimicrobial agents to which the isolate was resistant, and ‘b’ is the number of antimicrobial agents against which the isolate was tested [46]. An isolate resistant to at least one antimicrobial agent in three or more classes/categories of antimicrobial agents was considered multiple drug-resistant (MDR) [47].

#### 2.3.1. Detection of Methicillin-Resistant Strains

Resistance to methicillin by *S*. *aureus* isolates was assessed using the cefoxitin (30 µg) disc diffusion screening method in accordance with CLSI guidelines [45]. An isolate with an inhibition zone diameter ≤ 21 mm around the cefoxitin disc was considered a methicillin-resistant strain [45]. *S*. *aureus* ATCC 25923 was used as a quality-control strain for methicillin susceptibility.

#### 2.3.2. Detection of β-Lactamase Producing Strains

Production of the β-lactamase enzyme by *S*. *aureus* isolates with an inhibition zone diameter of ≥29 mm around the penicillin (10 units) disc was assessed using the penicillin zone-edge test method in accordance with the CLSI guidelines [45]. An isolate with a sharp zone edge/ “cliff” surrounding the penicillin disc was considered a β-lactamase-producer whereas any isolate with a fuzzy (“beach”-like) inhibition zone edge was considered β-lactamase-negative [45]. *S*. *aureus* ATCC 25923 was used as a quality-control strain for β-lactamase expression.

#### 2.3.3. Assay for Vancomycin Resistance

Resistance to vancomycin was determined with the agar screening method following CLSI guidelines [45]. Briefly, 10 µL of 0.5 McFarland’s turbidity colony suspension (1 × 10^8^ cfu/mL) of each isolate was spot-inoculated using a sterile wire loop on vancomycin agar (tryptic soy agar with 8 µg/mL vancomycin) and incubated at 37 °C for 18 h. An isolate with >1 colony on the agar indicated reduced susceptibility to vancomycin [45]. *S*. *aureus* ATCC 25923 was used as a quality-control strain for vancomycin susceptibility.

#### 2.3.4. Assay for Inducible Clindamycin Resistance

Phenotypic inducible clindamycin resistance of erythromycin- and clindamycin-resistant *S*. *aureus* isolates were assayed using the D-zone test method in accordance with the guidelines of the CLSI [45]. *S*. *aureus* ATCC 25923 was used as a quality-control strain for inducible clindamycin resistance.

### 2.4. Heavy Metal Tolerance

The tolerance of the *S*. *aureus* isolates to heavy metals was evaluated by a protocol previously described [48,49]. The heavy metals used in this assay include Copper sulphate pentahydrate (CuSO_4_·5H_2_O), Cadmium nitrate tetrahydrate (Cd(NO_3_)2·4H_2_O), Zinc sulfate (ZnSO_4_·7H_2_O), and Lead nitrate (PbNO_3_)_2_. Briefly, 10 µL of 1 × 10^8^ cfu/mL (0.5 McFarland turbidity standard) of each *S*. *aureus* isolate was spot-inoculated on Mueller-Hinton agar plates supplemented with increasing concentrations of each heavy metal salt. The starting concentration of the heavy metal was 50 µg/mL and the concentration was increased by 50 µg/mL at intervals until the isolate failed to grow. The higher concentration plates were inoculated with an isolate from the previous concentration and plates were incubated at 37 °C for 24–48 h. An isolate with growth on an agar plate was considered resistant/tolerant to the concentration of heavy metal [49]. An inhouse *S*. *aureus* strain resistant to the heavy metals was used as a quality-control strain for heavy metal resistance.

### 2.5. Detection of Virulence Potentials

#### 2.5.1. Haemolysin Production

Production of α-hemolysis (clear transparent zone around colonies) and β-hemolysis (partial greenish zone around colonies) was determined by spot-inoculating 10 µL of 1 × 10^8^ cfu/mL (0.5 McFarland turbidity standard) of each *S*. *aureus* isolate on blood agar (tryptic soy agar with 5% *v*/*v* sheep blood) and incubating at 37 °C for 24 h [33]. *S*. *aureus* ATCC 25923 was used as the positive control.

#### 2.5.2. Gelatinase Activity

Gelatinase activity was determined by spot-inoculating 10 µL of 1 × 10^8^ cfu/mL (0.5 McFarland turbidity standard) of each *S*. *aureus* isolate on gelatin agar (tryptic soy agar with 3% *w*/*v* gelatin) and incubating at 37 °C for 48 h [33,50]. The presence of a transparent halo surrounding the colonies following the flooding of the agar with Frazier solution (mercuric chloride 15 g, hydrochloric acid 37%, 20 mL distilled water, 100 mL) indicated gelatinase production [50]. *S*. *aureus* ATCC 25923 was used as a positive control.

#### 2.5.3. Casein Hydrolysis

Caseinase activity was established by spot-inoculating suspension of each isolate on caseinase agar (tryptic soy agar with 15% *w*/*v* soluble casein) and incubated at 37 °C for 24 h [33]. The presence of a clear transparent zone surrounding the growth on the flooding of the plate with Frazier solution indicated caseinase production. *Enterococcus faecalis* ATCC 29212 was used as a positive control.

#### 2.5.4. Lipase Activity

The ability of the isolates to break down lipids was determined following a previously described protocol [51]. Briefly, 10 µL of 1 × 10^8^ cfu/mL of each isolate was spot-inoculated on tween 80 agar (tryptic soy agar with 1% *v*/*v* tween 80) and incubated at 37 °C for 48 h. An isolate with a yellowish zone surrounding the growth was regarded as a lipase producer (Ramnath et al., 2017). *S*. *aureus* ATCC 25923 was used as a positive control.

#### 2.5.5. Amylase Production

The ability of the isolates to hydrolyze starch was determined per Al-Dhabi et al. [52] and Preda et al. [33]. Briefly, 10 µL of 1 × 10^8^ cfu/mL of each isolate was spot-inoculated on starch agar (tryptic soy agar with 1% *w*/*v* soluble starch) and incubated at 37 °C for 48 h. An isolate with a brownish-black halo surrounding the growth following flooding with Lugol’s iodine was considered an amylase producer [52].

#### 2.5.6. Biofilm Production

The expression of biofilm by the isolates was assessed following the protocol described by Lee et al. [53]. Briefly, 10 µL of 1 × 10^8^ cfu/mL of each isolate was inoculated into the Congo red broth (tryptic soy broth with 0.08% *v*/*v* phenol red and 0.3% sucrose *w*/*v*) and incubated at 37 °C for 48 h. An isolate that turned the Congo red broth to black was considered a biofilm producer whereas cultures of the strains that were negative for biofilm were yellow, red, or black-red in color [53]. *S*. *epidermidis* ATCC 35984 was used as a positive control.

## 3. Data Analysis

The results of the various tests were entered into Microsoft Excel TM (Microsoft, Redmond, WA, USA). Data on the occurrence of *S*. *aureus* and resistance were exported to SPSS v.15.0 (SPSS, Chicago, IL, USA) and Graph-Pad Prism statistical package v.8.3.1 (GraphPad Software, La Jolla, CA, USA) for analysis. The mean MARI of the isolates from each animal source was determined by adding up the MARI of all the isolates from that source and dividing by the number of isolates. The frequency, percentage and 95% confidence interval of variables were calculated as appropriate. The chi-squared χ^2^ [or Fisher’s exact test, where appropriate] test was used to determine the possible association between variables and the body site and between organisms and heavy metals.

## 4. Results

### 4.1. Occurrence of S. aureus and other Staphylococcus Species in Horses

Out of 180 horses sampled, 43 (23.9%) harbored *S*. *aureus*. From a total of 360 samples from the horses, 71 (19.7%) *S*. *aureus* and 30 (8.3%) other *Staphylococcus* species (spp.) were recovered (Table 1). *S*. *aureus* was isolated from 23.9% (43/180) nasal swabs and 15.6% (28/180) skin swabs. The skin of 28 out of the 43 horses that harbored *S*. *aureus* in the nostril was also colonized. All the *Staphylococcus*-positive samples grew either *S*. *aureus* or other *Staphylococcus* spp. None of the samples grew both *S*. *aureus* together with another *Staphylococcus* spp. There was a significant association (*p* < 0.05) between the prevalence of *S*. *aureus/*other *Staphylococcus* species and body sites. 

### 4.2. Occurrence of Methicillin-Resistant and Methicillin-Susceptible S. aureus

Out of the 71 *S*. *aureus* isolates, 2 (2.8%) exhibited resistance to cefoxitin and thus considered MRSA while the remaining 69 (97.2%) were MSSA (Table 2). The two MRSA strains were from different horses, thus a prevalence rate of 1.1% (2/180). The MRSA detection rates in specific body sites were 2.3% (1/43) in the nostril and 3.6% (1/28) on the skin. There was no significant difference (*p* > 0.05, χ^2^ = 0.999) between the proportions of MRSA recovered from the nostril and skin. 

### 4.3. Antimicrobial Resistance Profile S. aureus Isolates

**Figure 1:**
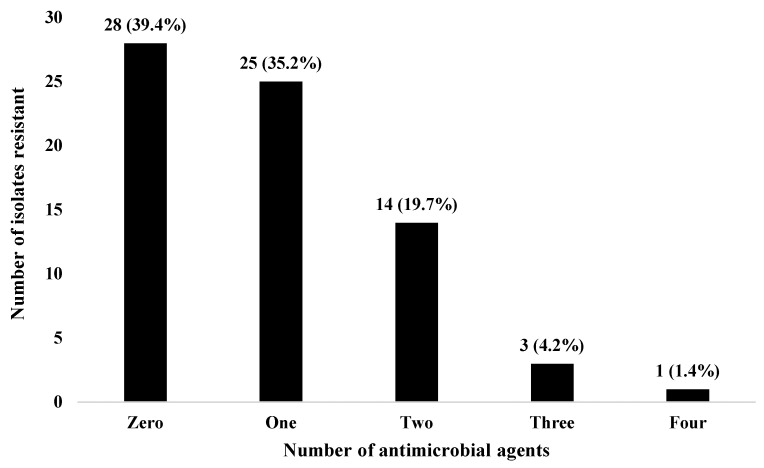
Resistance to antimicrobial agents among 71 *Staphylococcus aureus* from horses slaughtered in Obollo-Afor, Southeast Nigeria.

The antimicrobial resistance profile of the 71 *S*. *aureus* isolates to 11 antimicrobial agents (Table 3) revealed that 53.5% were resistant to penicillin while 16.9%, 8.5% and 5.6% were resistant to erythromycin, rifampicin and tetracycline, respectively. The least resistance was demonstrated against trimethoprim-sulphamethoxazole (4.2%) and cefoxitin (2.8%). Only 43 of the 71 strains consisting of 25 of 43 nasal isolates and 18 of 28 skin isolates, exhibited resistance to antimicrobial agents. None of the isolates were resistant to ciprofloxacin, linezolid, vancomycin, gentamicin, and clindamycin. Among the 71 isolates, 39.4% were susceptible to all the antimicrobial agents whereas 35.2%, 19.7%, 4.2% and 1.4% were resistant to one, two, three and four antimicrobial agents, respectively (Figure 1). Out of the 71 strains, 18 (25.3%) exhibited multiple drug resistance (resistance to two or more antimicrobial agents). The 43 resistant isolates exhibited 11 multiple antimicrobial resistance patterns (resistance to two or more antimicrobial agents) with PEN-ERY being the predominant (*n* = 5) (Table 4). Four (9.3%) of the 43 isolates were MDR. The mean MARI of the 43 isolates was 0.24 (range 0.09–0.36). 

#### 4.3.1. Vancomycin and Inducible Clindamycin Resistance of *S. aureus* Isolates

None of the 71 *S*. *aureus* isolates was resistant to vancomycin and clindamycin.

#### 4.3.2. β-Lactamase Production by *S. aureus* Isolates

Out of the 71 *S*. *aureus* isolates, 5 (7.0%) were β-lactamase-producers. The five strains consist of 3 (7.0%) of the 43 nostril isolates and 2 (7.1%) of the 28 skin isolates.

### 4.4. Virulence Potentials of Isolates

**Figure 2:**
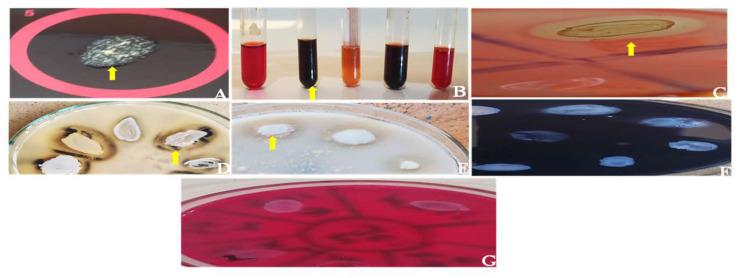
Phenotypic tests detecting virulence potentials of *Staphylococcus aureus* isolates from horses in Nigeria. Clumping factor expression test using Staphytect Plus™ latex agglutination kit (**A**), biofilm production test using 0.03% Congo red and 0.8% sucrose tryptic soy broth (**B**), haemolysin production test using 5% sheep blood agar (**C**), gelatinase production test using 3% gelatin agar (**D**), caseinase test using 1% casein agar (**E**), amylase production test using 10% starch agar (**F**), and lipase production test using 1% tween 80 and 0.01% phenol red agar (**G**). The arrow indicates a positive result.

All 71 isolates phenotypically expressed virulence factors. Among them, all (100%) expressed clumping factor and catalase, 38 (53.5%) showed gelatinase (Gel) production, 31 (43.7%) showed hemolysis, 17 (23.9%) portrayed biofilm production, and 13 (18.3%) displayed caseinase (Cas) activity (Table 5) (Figure 2). Asides from clumping factor (Clf) and catalase (Cat), the nostril MRSA strain expressed only gelatinase while the skin MRSA strain displayed hemolysis and gelatinase. None of the isolates produced amylase and lipase, and none of them was simultaneously positive for all the tested virulence factors. The 71 isolates expressed 11 potential virulence patterns with Clf-Cat-Hyl-Gel (*n* = 18) being the predominant pattern (Table 6). There was no significant association (*χ*^2^ = 24.00; *p* = 0.24) between the virulence factors and the body site.

### 4.5. Heavy Metal Tolerance of S. aureus Isolates

All the 71 *S*. *aureus* isolates were tested for heavy metal susceptibility against four heavy metals with increasing concentrations up to a concentration of 1500 µg/mL. Moderate to high resistance rates were found against the different heavy metals with 39.4%, 50.7%, 49.3%, and 60.6% of the isolates resistant to 1500 µg/mL concentration of Cd, Cu, Pb, and Zn, respectively (Table 7). From 100 to 1500 µg/mL, the isolates exhibited more zinc tolerance than other heavy metals tested. There was no significant difference (*p* > 0.05) between the isolates and different concentrations of the heavy metals. 

## 5. Discussion

The 23.9% occurrence of *S*. *aureus* in this study indicates that a sizeable percentage of horses slaughtered in Obollo-Afor, Southeast Nigeria are colonized by *S*. *aureus*. Significantly higher recovery of *S*. *aureus* than other *Staphylococcus* spp. from the 360 samples suggests that *S*. *aureus* is the predominant staphylococcal species colonizing horses in the study area. The closeness of humans to these horses (in their stables) until slaughter might be responsible for the high colonization by *S*. *aureus*. Interaction with anthropogenic- and agricultural-impacted environments could also be postulated. Significantly, a higher occurrence of *S*. *aureus* in the nostril (23.9%) than on the skin (15.6%) suggests that the horses were colonized more in the nostrils than on the skin. This finding is not surprising as the anterior nares of mammals, including horses, are established to be the main reservoir/ecological niche for *S*. *aureus* [54,55]. The presence of *S*. *aureus* in the nostril and skin of slaughtered horses is a food safety/public health concern as handlers of these animals could easily get colonized/infected by the organism and subsequently serve as vehicles for its transmission to their household and the public. Cross-contamination of the slaughterhouse environment and horse meat from the skin and nostrils is also easy in Nigerian slaughterhouses because unhygienic slaughter techniques are employed in these slaughterhouses. In the slaughterhouses, the butchers do not wear personal protective equipment (PPE) such as gloves and nose masks and they touch the horses, their carcasses and skin processed in open-air slabs, with bare hands. Moreover, the skin of the horses is flayed on these contaminated slabs and frequently touched by the butchers and potential buyers, most of whom consume the horse skin. Since *S*. *aureus* produces enterotoxins, handling and consumption of raw and undercooked horse meat contaminated by *S*. *aureus* could result in food-borne disease outbreaks such as staphylococcal food poisoning. Although *S*. *aureus* is a normal inhabitant of the nasal cavity and skin of horses [2], and the risk factor for colonization of the horses by *S*. *aureus* was not evaluated in this study, possible exogenous sources of the *S*. *aureus* in the horses include cross-contamination from fomites (grooming gears and stable environment) and horses during their transportation from the northern to the southeastern region, contaminated hands of the handlers, flies that perched on the horses serving as vectors, and/or contaminated grasses/herbages that the horses contacted. 

The 23.9% nasal swab *S*. *aureus* occurrence in this study is higher than the 20.3%, 19.5%, 2.0%, 7.9%, 6.8% and 13.5% nasal swab *S*. *aureus* occurrence among healthy horses in the USA [13], South Korea [36], Iran [37], Canada [56], Germany [42] and Denmark [10]. However, it is lower than 40% *S*. *aureus* occurrence among horses in Belgium [55]. Differences in the *S*. *aureus* occurrence in the studies is due to variation in the method of detection (whether phenotypic or molecular) of *S*. *aureus* strains, health status of the animals, equine husbandry practices, environmental factors, and degree of contamination by *S*. *aureus*. Notably, this study’s 15.6% groin skin colonization by *S*. *aureus* is considerably high. Although non-nasal site colonization of *S*. *aureus* in animals is established [1,2], the presence of *S*. *aureus* on the skin of slaughtered horses calls for attention because individuals such as veterinarians, sellers, butchers, meat retailers, and buyers that are in direct contact with these horses and associated products could easily acquire *S*. *aureus* from the animals. Moreover, those that handle/process the hides from these horses colonized by *S*. *aureus* on the skin can easily contract the organism. Therefore, education of the horse handlers (jockeys, sellers, butchers, and so on) on the use of PPE and hand hygiene when handling these animals is warranted [55].

Methicillin resistance is a serious public health threat since MRSA is associated with a high rate of morbidity, hospitalization cost, and mortality in humans and animals [1,57]. In this study, the MRSA carriage rate among horses was 1.1% (2/180). This MRSA occurrence rate suggests that a low percentage of horses slaughtered in southeastern Nigeria constitute reservoirs for MRSA. The absence of a statistically significant difference between the proportions of MRSA from the nostrils and skin of the horses, suggests that both nasal and non-nasal sites of horses in the study area could be similarly colonized by MRSA. The 1.1% MRSA carriage rate among horses in this study is higher than the 0.5% MRSA carriage rate among horses at the farm level in the USA [13]. The MRSA carriage rate in horses in Canada was reported to range between 3–13% [12,56,58]. An Australian study recorded a 3.7% MRSA carriage rate [35]. Previous studies in horses from European countries reported MRSA carriage rates in horses ranging between 0.6–40% [10,41,42,43,55,59,60,61] while studies from Asia reported MRSA carriage rates in horses ranging between 1.7–8.7% [36,37,39]. Nonetheless, the result of this present study (1.1%) is in stark contrast to previous studies by Burton et al. [56] and Ryhhoud et al. [34] who did not recover MRSA from horses. It is worth noting that Okorie-Kanu et al. [9] reported a 1.5% prevalence rate of MRSA in pigs in Enugu State southeastern Nigeria, Odetokun et al. [8] recorded a 1.1% prevalence of MRSA in ready-to-slaughter pigs in Ibadan, Oyo State Southwestern Nigeria. In contrast, Momoh et al. [62] did not detect MRSA in pigs from Jos, North Central, Nigeria. 

In the present study, 2.8% of the 71 *S*. *aureus* were MRSA suggesting low selection against methicillin/oxacillin. This low methicillin resistance is attributable to the fact that methicillin/oxacillin/cefoxitin was very likely not used in treating the horses. Methicillin/oxacillin has never been available/used in any sector in Nigeria, and cefoxitin though available for human medicine, is not known to be used in the animal sector in Nigeria. Nevertheless, other β-lactam antibiotics such as penicillin, ampicillin, amoxicillin, cloxacillin, and cephalosporins are frequently used in human and animal medicine in Nigeria. Therefore, the use of these antibiotics might have exerted the selection pressure for methicillin resistance possibly through the acquisition of *mec*A/C genes or other mechanisms [63,64]. The 2.8% MRSA detection rate in this study is lower than 6.1–19.4% MRSA among *S*. *aureus* from horses elsewhere [36,37,40,65]. Variation in the results of the studies is probably due to differences in sampling methods, type of sample analyzed, MRSA detection method (whether phenotypic or molecular), degree of contamination of horses’ environment and colonization, antimicrobial usage, management of the animals at farms, abattoir and prior to slaughter in the study areas. It is worth noting that Sanda and Idris [44] used cefoxitin and oxacillin disc methods and reported an 8.0% MRSA detection rate among 125 *S*. *aureus* from recreational horses in Kano State Northwestern, Nigeria. Thus, shedding of *S*. *aureus*/MRSA prior to sampling (such as during grooming) of the horses is postulated.

Low to moderate rates of resistance to erythromycin (16.9%), rifampicin (8.5%), tetracycline (5.6%), trimethoprim-sulphamethoxazole (4.2%), and penicillin (53.5%) were observed among the isolates investigated in this study. Although the medical history of the horses could not be assessed since they were from unknown sources in northern Nigeria, the resistance rates could be due to the low usage of these antimicrobials in the management of the horses. Moreover, these horses are meant for slaughter and barely receive medical treatment (since they stay for a short time) in the study area. Nevertheless, the penicillin resistance could be due to β-lactamase production or other β-lactam resistance mechanisms as well as collateral resistance conferred by methicillin resistance in one of the MRSA [63]. β-lactamase is the major enzymatic mechanism of penicillin resistance in *S*. *aureus* and MRSA is well known to exhibit co-resistance to other classes of antimicrobial agents [63]. Although at low rates, ERY (16.9%) and RIF (8.5%) resistance in this study suggested selection against macrolides and ansamycins. This finding is of concern because macrolides (ERY) and ansamycins (RIF) are critically-important antibiotics for human medicine [66,67]. Although not commonly used to treat staphylococcal infections, ERY combined with RIF is widely used in treating other equine bacterial infections, such as pneumonia attributable to *Rhodococcus* species [40,68,69]. SXT resistance in this study is 4.2% suggesting low SXT selection pressure. SXT is commonly used to treat infections in horses as it provides broad-spectrum antimicrobial coverage [40]. Elsewhere, 29.6–86.1% ERY and 100% SXT resistance was observed among equine *S*. *aureus* isolates [36,41,70], suggesting that low ERY, RIF and SXT usage could be the reason for the low selection in this present study. 

The low multidrug resistance (9.3%) observed in this study further suggests that the sampled horses were not previously managed/treated with many antimicrobials. Concordantly, a lesser proportion (18/71, 25.4%) of the isolates in this study exhibited multiple resistance (resistance to two or more antibiotics) and only one of the MRSA was a multiple drug-resistant strain. Moreover, a MARI > 0.2 indicates a ‘high-risk’ source of contamination [46], and the mean MARI of isolates from horses in this study was 0.24. The absence of clindamycin and vancomycin resistance in this study may be because clindamycin is contraindicated in horses due to its potential to cause fatal enterocolitis complications [40]. Since clindamycin and vancomycin are critical in the management of *S*. *aureus* infections, it would be desirable that this favorable situation (lack of clindamycin and vancomycin resistance) is preserved through the establishment of antimicrobial stewardship programs.

The high heavy metal tolerance in this study suggests selection against the metals. The lack of a significant difference in the resistance of the isolates to the heavy metals indicates that the isolates were similarly exposed to the metals, and resistance to them is conferred by similar mechanisms [28]. Nonetheless, the higher selection against zinc than other heavy metals may be because zinc is often used as a livestock feed supplement and often found in the environment [29]. However, the finding of moderate to high heavy metal resistance in this study calls for attention because the development of antimicrobial and heavy metal-resistant bacteria, especially *S*. *aureus*, complicates treatment and poses major threats to human and animal health and in medical/veterinary practice [49]. Moreover, resistance to antibiotics and heavy metals can be easily/rapidly disseminated by staphylococci through mobile genetic elements [71]. The selection pressure for heavy metal resistance could be from the use of detergents in washing, feeding and drinking troughs [49]. Consumption of and/or contact with herbage and/or water contaminated with heavy metal originating from fertilizers used in crop farms and mine ponds prevalent in northern Nigeria [72,73], could also be the sources of the selection pressure. 

*S*. *aureus* is the commonest cause of hospital (human/veterinary)-associated infections and death worldwide [4]. Therefore, the expression of VFs by *S*. *aureus* is a major public health concern. In this study, all the isolates possessed virulence potentials having produced one or more of the tested virulence factors. All isolates in this work are typical *S*. *aureus,* having produced catalase and clumping factor that help *S*. *aureus* to form biofilm and evade the host immune responses [32,33]. A considerable percentage (23.9%) of the isolates in this study were biofilm producers, indicating that these strains can be recalcitrant to antimicrobial therapy when they infect individuals. Little et al. [40] reported an 11% occurrence rate of biofilm-producers among *S*. *aureus* isolates from equids in the USA but the methods used differed. In this experiment, the Congo red broth test which correlates with the carriage of genes encoding biofilm was employed [53]. Biofilm production enables *S*. *aureus* to survive harsh environmental conditions and cause chronic infection in individuals with indwelling devices (such as catheters and pacemakers), evade the immune system, and resist the effect of antibiotics and disinfectants [33,53,74,75]. Gelatinase is a metalloproteinase that can cleave hemoglobin, fibrinogen, fibronectin, gelatin, collagen, laminin, and numerous peptides/proteins thereby promoting the dissemination of the organism and it also helps in biofilm formation [33]. Thus, it is of public health concern that a high proportion (53.5%) of isolates in this study were gelatinase producers. The α-haemolysin is an exotoxin produced by *S*. *aureus* that destroys blood cells, thereby enhancing the invasion of the organism [32,33]. In this study, a high proportion (43.7%) of the isolates were α-hemolytic (complete hemolysis) phenotype, and can thus cause serious infections. Caseinase is a protease and proteases are a significant class of biomolecules that cleaves peptide bonds [76,77]. Unfortunately, a sizeable percentage (18.3%) of isolates in this study displayed caseinase activity. More worrisome is that the isolates exhibited 11 virulence patterns, meaning they could express a diversity of VFs, and the multiple drug-resistant strains, including the MRSA, also possessed these virulence potentials. This indicates that the isolates could potentially cause difficult-to-treat diseases in individuals infected by them. 

This work has some limitations. The number of samples processed is not large enough to conclude that the MRSA prevalence in slaughtered horses in the study is as low as observed in this study. Horse meat samples were not examined; thus, the public health importance of *S*. *aureus* colonization of slaughtered horses was not fully showcased. Molecular characterization of the isolates that would help understand the phenotypic antimicrobial/heavy metal resistance and virulence potentials was not done. Therefore, the absence of any trait (resistance or virulence potential) by some isolates in this study, did not preclude them from being resistant and/or virulent. 

## 6. Conclusions

This study has shown that a considerable percentage of horses slaughtered in Obollo-Afor Southeastern Nigeria are potential reservoirs of virulent multiple drug-resistant and heavy metal-tolerant *S*. *aureus* with a low proportion of the horses serving as a potential source for the transmission of MRSA to humans and the environment. The presence of multiple drug-resistant and heavy metal-tolerant *S*. *aureus* in hobby and food animals, as observed in this study, is a food safety concern and portends danger to the health of individuals handling horses and/or consuming horse meat/skin in the study area because these are organisms capable of causing food-borne disease outbreaks and jeopardizing antimicrobial therapy in infected individuals. They are also able to breach biosecurity lines in farms and households to cause serious infections in humans and animals in the study area. The spread of multiple-drug-resistant and heavy metal-tolerant *S*. *aureus* could hugely impact the ecology and epidemiology of antimicrobial resistance in Nigeria. Therefore, since *S*. *aureus* is the leading cause of death globally, attention should be paid to the hygiene of horse slaughterhouses and their workers in Obollo-Afor Southeastern Nigeria, and the use of antimicrobial agents and heavy metal supplements in equine/livestock husbandry in Nigeria to curb the risk of farm/stable-to-plate transmission of multiple drug- and heavy metal-resistant *S*. *aureus* along the horse meat chain.

## Figures and Tables

**Table 1:** Prevalence of *Staphylococcus* in slaughtered horses at Obollo-Afor, Southeast Nigeria.

Sample	Number of Samples Processed	Total *Staphylococcus* Isolated	*S*. *aureus*	Other *Staphylococcus* Species	*p* Value	Odds Ratio	95% Confidence Interval
Skin swab	180	40 (22.2)	28 (15.6)	12 (6.7)	0.0007	5.4	2.01–13.26
Nasal swab	180	61 (33.8)	43 (23.9)	18 (10)	0.0016	3.7	1.64–8.33
Total	360	101 (28.2)	71 (19.7)	30 (8.3)			

**Table 2:** Detection rate of methicillin-resistant *Staphylococcus aureus* in slaughtered horses at Obollo-Afor, Southeast Nigeria.

Sample	*S*. *aureus*	MRSA (%)	MSSA (%)	*p* Value	Odds Ratio	95% CI
Nasal swab	43	1 (2.3)	42 (97.7)	0.999	0.64	0.03–12.63
Skin swab	28	1 (3.6)	27 (96.4)	0.999	0.64	0.03–12.63

MRSA = methicillin-resistant *S*. *aureus*, MSSA = methicillin-susceptible *S*. *aureus*, CI = confidence interval.

**Table 3:** Antimicrobial susceptibility profile of *Staphylococcus aureus* from horses slaughtered in Obollo-Afor Southeast, Nigeria.

Antimicrobial Class	Antimicrobial Agent	Number (%) of Isolates Resistant (N = 71)		
		Nasal Cavity (*n* = 43)	Groin (*n* = 28)	Total (%)
Folate pathway antagonists	Trimethoprim-sulphamethoxazole	0	3	3 (4.2)
Fluoroquinolones	Ciprofloxacin	0	0	0 (0.0)
Tetracyclines	Tetracycline	2	2	4 (5.6)
Oxazolidinones	Linezolid	0	0	0 (0.0)
Ansamycins	Rifampicin	4	2	6 (8.5)
β-lactams	Penicillin	24	14	38 (53.5)
	Cefoxitin	1	1	2 (2.8)
Macrolides	Erythromycin	6	6	12 (16.9)
Glycopeptides	Vancomycin	0	0	0 (0.0)
Aminoglycosides	Gentamicin	0	0	0 (0.0)
Lincosamides	Clindamycin	0	0	0 (0.0)

N = total number of isolates, *n* = number of isolates.

**Table 4:** Antimicrobial resistance patterns and multiple antimicrobial resistance indices of *Staphylococcus aureus* from horses slaughtered in Obollo-Afor Southeast, Nigeria.

SN	No. of Antimicrobials (MARI)	Antimicrobial Resistance Pattern	Nostril (N = 25)	Skin (N = 18)	Total (N = 43)	No. of Antimicrobial Classes	No. (%) of MDR Strains
1	1 (0.09)	ERY	1	1	2	1	
2		PEN	15	7	22		
3		RIF	0	1	1		
4	2 (0.18)	RIF-PEN	2	0	2	2	
5		PEN-ERY	2	3	5		
6		PEN-CFT	1	0	1		
7		TET-PEN	1	1	2		
8		PEN-GEN	0	1	1		
9		RIF-ERY	0	1	1		
10		SXT-TET	0	1	1		
11		SXT-PEN	0	1	1		
12	3 (0.27)	RIF-PEN-ERY	2	0	2	3	4 (9.3)
13		TET-PEN-ERY	1	0	1		
14	4 (0.36)	SXT-PEN-CFT-ERY	0	1	1	3	

N = number of isolates, MDR = multidrug-resistant, MARI = multiple antimicrobial resistance index, SXT = trimethoprim-sulphamethoxazole, ERY = erythromycin, CFT = cefoxitin, PEN = penicillin, TET = tetracycline, RIF = rifampicin, GEN = gentamicin.

**Table 5:** Frequency of phenotypic virulence factors expressed by *Staphylococcus aureus* from horses slaughtered in Obollo-Afor, Southeast Nigeria.

Virulence Factor	Number of Isolates		Total (N = 71)	% Frequency
	Nasal Cavity (N = 43)	Groin (N = 28)		
Clumping factor	43	28	71	100
Catalase	43	28	71	100
Haemolysin	19	12	31	43.7
Biofilm	10	7	17	23.9
Gelatinase	24	14	38	53.5
Casienase	7	6	13	18.3
Amylase	0	0	0	0.0
Lipase	0	0	0	0.0

N = number of isolates.

**Table 6:** Phenotypic virulence patterns of *Staphylococcus aureus* from horses slaughtered in Obollo-Afor Southeast Nigeria.

S/N	Phenotypic Virulence Pattern	Number of Isolates		Total (N = 71)	% Frequency
		Nasal Cavity (N = 43)	Groin (N = 28)		
1.	Clf-Cat-Hyl-Bfm-Gel-Cas	3	2	5	7.0
2.	Clf-Cat-Hyl-Gel	13	5	18	25.4
3.	Clf-Cat-Hyl-Bfm-Gel	9	3	12	16.9
4.	Clf-Cat-Gel	3	2	5	7.0
5	Clf-Cat-Bfm-Gel	3	3	6	8.5
6.	Clf-Cat-Gel-Cas	3	1	4	5.6
7.	Clf-Cat-Hyl	0	5	5	7.0
8.	Clf-Cat-Hyl-Cas	2	0	2	2.8
9.	Clf-Cat-Hyl-Bfm	2	0	2	2.8
10.	Clf-Cat-Hyl-Gel-Cas	5	5	10	14.1
11.	Clf-Cat-Bfm-Cas	0	2	2	2.8

N = Number of isolates, Clf = clumping factor, Cat = catalase, Hyl = haemolysin, Bfm = biofilm, Gel = gelatinase, Cas = caseinase.

**Table 7:** Prevalence of *Staphylococcus aureus* from horses growing at increasing heavy metal concentrations.

Concentration	Number (%) of Isolates that Grew on Agar Containing Heavy Metal (N = 71)				*χ*^2^ Value	*p* Value
	Cadmium	Copper	Lead	Zinc		
50 µg/mL	42 (59.2%)	50 (70.4%)	44 (62.0%)	50 (70.4%)	8.00 ^a^	0.24
100 µg/mL	40 (56.3%)	42 (59.2%)	44 (62.0%)	50 (70.4%)	12.00 ^a^	0.21
500 µg/mL	39 (54.9%)	38 (53.5%)	36 (50.7%)	47 (66.2%)	12.00 ^a^	0.21
1000 µg/mL	28 (39.4%)	38 (53.5%)	35 (49.3%)	45 (63.4%)	12.00 ^a^	0.21
1500 µg/mL	28 (39.4%)	36 (50.7%)	35 (49.3%)	43 (60.6%)	12.00 ^a^	0.21

^a^ superscript across rows indicates a significant difference, N = number of isolates.

## Data Availability

Not applicable.

## References

[1] Aires-de-Sousa M. (2017). Methicillin-Resistant *Staphylococcus aureus* among Animals: Current Overview. Clin. Microbiol. Infect..

[2] Haag A.F., Fitzgerald J.R., Penadés J.R. (2019). *Staphylococcus aureus* in Animals. Microbiol. Spectr..

[3] Murray C.J., Ikuta K.S., Sharara F., Swetschinski L., Robles Aguilar G., Gray A., Han C., Bisignano C., Rao P., Wool E. (2022). Global Burden of Bacterial Antimicrobial Resistance in 2019: A Systematic Analysis. Lancet.

[4] Ikuta K.S., Swetschinski L.R., Robles Aguilar G., Sharara F., Mestrovic T., Gray A.P., Davis Weaver N., Wool E.E., Han C., Gershberg Hayoon A. (2022). Global Mortality Associated with 33 Bacterial Pathogens in 2019: A Systematic Analysis for the Global Burden of Disease Study 2019. Lancet.

[5] WHO (2017). Global Priority List of Antibiotic-Resistant Bacteria to Guide Research, Discovery, and Development of New Antibiotics. Cadernos Pesqui..

[6] Alhmidi H., Cadnum J.L., Koganti S., Jencson A.L., Rutter J.D., Bonomo R.A., Wilson B.M., Mayer J., Samore M.H., Donskey C.J. (2019). Shedding of Methicillin-Resistant *Staphylococcus aureus* by Colonized Patients during Procedures and Patient Care Activities. Infect. Control Hosp. Epidemiol..

[7] Anyanwu M.U., Ugwu I.C., Onah C.U. (2018). Occurrence and Antibiogram of Generic Extended-Spectrum Cephalosporin-Resistant and Extended-Spectrum β-Lactamase-Producing Enterobacteria in Horses. Maced. Vet. Rev..

[8] Odetokun I.A., Maurischat S., Adetunji V.O., Fetsch A. (2022). Methicillin-Resistant *Staphylococcus aureus* from Municipal Abattoirs in Nigeria: Showing Highly Similar Clones and Possible Transmission from Slaughter Animals to Humans. Foodborne Pathog. Dis..

[9] Okorie-Kanu O.J., Anyanwu M.U., Ezenduka E.V., Mgbeahuruike A.C., Thapaliya D., Gerbig G., Ugwuijem E.E., Okorie-Kanu C.O., Agbowo P., Olorunleke S. (2020). Molecular Epidemiology, Genetic Diversity and Antimicrobial Resistance of *Staphylococcus aureus* Isolated from Chicken and Pig Carcasses, and Carcass Handlers. PLoS ONE.

[10] Islam M.Z., Espinosa-Gongora C., Damborg P., Sieber R.N., Munk R., Husted L., Moodley A., Skov R., Larsen J., Guardabassi L. (2017). Horses in Denmark Are a Reservoir of Diverse Clones of Methicillin-Resistant and -Susceptible *Staphylococcus aureus*. Front. Microbiol..

[11] Anderson M.E.C. (2008). Methicillin-Resistant Staphylococcus aureus in Horses: Aspects of Detection, Clinical Infection and the Potential for Zoonotic Transmission.

[12] Weese J.S., Rousseau J., Traub-Dargatz J.L., Willey B.M., McGeer A.J., Low D.E. (2005). Community-Associated Methicillin-Resistant *Staphylococcus aureus* in Horses and Humans Who Work with Horses. J. Am. Vet. Med. Assoc..

[13] McElwee M., Griffith S., Mediavilla J., Kreiswirth B., Voyich J., Moreaux S. (2009). Characterization of *Staphylococcus aureus* in Horses and Horse Personnel in Southwest Montana. J. Equine Vet. Sci..

[14] Pantosti A. (2012). Methicillin-Resistant *Staphylococcus aureus* Associated with Animals and Its Relevance to Human Health. Front. Microbiol..

[15] Cuny C., Witte W. (2013). Livestock Associated MRSA Detected from Livestock: The Impact on Humans. Fleischwirtschaft.

[16] Álvarez–Narváez S., Berghaus L.J., Morris E.R.A., Willingham-Lane J.M., Slovis N.M., Giguere S., Cohen N.D. (2020). A Common Practice of Widespread Antimicrobial Use in Horse Production Promotes Multi-Drug Resistance. Sci. Rep..

[17] Isgren C. (2018). Antimicrobial Resistance in Horses. Vet. Rec..

[18] Isgren C.M. (2021). Improving Clinical Outcomes via Responsible Antimicrobial Use in Horses. Equine Vet. Educ..

[19] Anyanwu M.U., Jaja I.F., Nwobi O.C., Mgbeahuruike A.C., Ikpendu C.N., Okafor N.A., Oguttu J.W. (2022). Epidemiology and Traits of Mobile Colistin Resistance (Mcr) Gene-Bearing Organisms from Horses. Microorganisms.

[20] Anyanwu M.U., Okorie-Kanu O.J., Ogugua A.J., Ezenduka E.V., Anidebe C.O. (2019). Occurrence, Antibiogram and Vancomycin Resistance of Generic Enterococci in Horses in Nigeria. Rev. Med. Vet..

[21] Turner N.A., Sharma-Kuinkel B.K., Maskarinec S.A., Eichenberger E.M., Shah P.P., Carugati M., Holland T.L., Fowler V.G. (2019). Methicillin-Resistant *Staphylococcus aureus*: An Overview of Basic and Clinical Research. Nat. Rev. Microbiol..

[22] Anafo R.B., Atiase Y., Kotey F.C.N., Dayie N.T.K.D., Tetteh-Quarcoo P.B., Duodu S., Osei M.M., Alzahrani K.J., Donkor E.S. (2021). Methicillin-Resistant *Staphylococcus aureus* (MRSA) Nasal Carriage among Patients with Diabetes at the Korle Bu Teaching Hospital. PLoS ONE.

[23] Lee B.Y., Singh A., David M.Z., Bartsch S.M., Slayton R.B., Huang S.S., Zimmer S.M., Potter M.A., Macal C.M., Lauderdale D.S. (2013). The Economic Burden of Community-Associated Methicillin-Resistant *Staphylococcus aureus* (CA-MRSA). Clin. Microbiol. Infect..

[24] Uematsu H., Yamashita K., Kunisawa S., Fushimi K., Imanaka Y. (2017). Estimating the Disease Burden of Methicillin-Resistant *Staphylococcus aureus* in Japan: Retrospective Database Study of Japanese Hospitals. PLoS ONE.

[25] Timsina R., Shrestha U., Singh A., Timalsina B. (2021). Inducible Clindamycin Resistance and Erm Genes in *Staphylococcus aureus* in School Children in Kathmandu, Nepal. Future Sci. OA.

[26] Amin A.N., Cerceo E.A., Deitelzweig S.B., Pile J.C., Rosenberg D.J., Sherman B.M. (2014). Hospitalist Perspective on the Treatment of Skin and Soft Tissue Infections. Mayo Clin. Proc..

[27] Aslam B., Khurshid M., Arshad M.I., Muzammil S., Rasool M., Yasmeen N., Shah T., Chaudhry T.H., Rasool M.H., Shahid A. (2021). Antibiotic Resistance: One Health One World Outlook. Front. Cell. Infect. Microbiol..

[28] Bazzi W., Abou Fayad A.G., Nasser A., Haraoui L.P., Dewachi O., Abou-Sitta G., Nguyen V.K., Abara A., Karah N., Landecker H. (2020). Heavy Metal Toxicity in Armed Conflicts Potentiates AMR in A. Baumannii by Selecting for Antibiotic and Heavy Metal Co-Resistance Mechanisms. Front. Microbiol..

[29] Nguyen C.C., Hugie C.N., Kile M.L., Navab-Daneshmand T. (2019). Association between Heavy Metals and Antibiotic-Resistant Human Pathogens in Environmental Reservoirs: A Review. Front. Environ. Sci. Eng..

[30] Cheung G.Y.C., Bae J.S., Otto M. (2021). Pathogenicity and Virulence of *Staphylococcus aureus*. Virulence.

[31] Zainulabdeen S.M.S., Dakl A.A. (2021). Review Article Pathogenicity and Virulence Factors in *Staphylococcus aureus*. Almuthanna J. Pure Sci..

[32] Singh V., Phukan U.J. (2019). Interaction of Host and *Staphylococcus aureus* Protease-System Regulates Virulence and Pathogenicity. Med. Microbiol. Immunol..

[33] Preda M., Mihai M.M., Popa L.I., Diţu L.M., Holban A.M., Manolescu L.S.C., Popa G.L., Muntean A.A., Gheorghe I., Chifiriuc C.M. (2021). Phenotypic and Genotypic Virulence Features of Staphylococcal Strains Isolated from Difficult-to-Treat Skin and Soft Tissue Infections. PLoS ONE.

[34] Rynhoud H., Meler E., Gibson J.S., Price R., Maguire T., Farry T., Bennett E., Hartono J., Soares Magalhães R.J. (2021). Epidemiology of Methicillin Resistant Staphylococcus Species Carriage in Companion Animals in the Greater Brisbane Area, Australia. Res. Vet. Sci..

[35] Axon J.E., Carrick J.B., Barton M.D., Collins N.M., Russell C.M., Kiehne J., Coombs G. (2011). Methicillin-Resistant *Staphylococcus aureus* in a Population of Horses in Australia. Aust. Vet. J..

[36] Choi S.-K., Hwang J.-Y., Park C.-S., Cho G.-J. (2018). Frequencies and Antimicrobial Susceptibility of Methicillin-Resistant *Staphylococcus aureus* (MRSA) Isolated from Horses in South Korea. Open Agric. J..

[37] Dastmalchi Saei H., Safari E. (2019). Methicillin Resistance and Clonal Diversity of *Staphylococcus aureus* Isolated from Nasal Samples of Healthy Horses in Iran. Ann. Microbiol..

[38] Zunita Z., Bashir A., Hafizal A. (2008). Occurrence of Multidrug Resistant *Staphylococcus aureus* in Horses in Malaysia. Vet. World.

[39] Tirosh-Levy S., Steinman A., Carmeli Y., Klement E., Navon-Venezia S. (2015). Prevalence and Risk Factors for Colonization with Methicillin Resistant *Staphylococcus aureus* and Other Staphylococci Species in Hospitalized and Farm Horses in Israel. Prev. Vet. Med..

[40] Little S.V., Hillhouse A.E., Lawhon S.D., Bryan L.K. (2021). Analysis of Virulence and Antimicrobial Resistance Gene Carriage in *Staphylococcus aureus* Infections in Equids Using Whole-Genome Sequencing. mSphere.

[41] Parisi A., Caruso M., Normanno G., Latorre L., Miccolupo A., Fraccalvieri R., Intini F., Manginelli T., Santagada G. (2017). High Occurrence of Methicillin-Resistant *Staphylococcus aureus* in Horses at Slaughterhouses Compared with Those for Recreational Activities: A Professional and Food Safety Concern?. Foodborne Pathog. Dis..

[42] Kaspar U., von Lützau K., Schlattmann A., Rösler U., Köck R., Becker K. (2019). Zoonotic Multidrug-Resistant Microorganisms among Non-Hospitalized Horses from Germany. One Health.

[43] Mallardo K., Nizza S., Fiorito F., Pagnini U., de Martino L. (2013). A Comparative Evaluation of Methicillin-Resistant Staphylococci Isolated from Harness Racing-Horses, Breeding Mares and Riding-Horses in Italy. Asian Pac. J. Trop. Biomed..

[44] Sanda M.I., Idris A.M. (2021). Nasopharyngeal Carriage of *Staphylococcus aureus* among Horses and Horse Handlers in Kano Metropolis, Nigeria. UMYU J. Microbiol. Res. (UJMR).

[45] Clinical and Laboratory Standards Institute (CLSI) (2021). Performance Standards for Antimicrobial Susceptibility Testing. CLSI Supplement M100.

[46] Krumperman P.H. (1983). Multiple Antibiotic Resistance Indexing of Escherichia Coli to Identify High-Risk Sources of Fecal Contamination of Foods. Appl. Environ. Microbiol..

[47] Magiorakos A.P., Srinivasan A., Carey R.B., Carmeli Y., Falagas M.E., Giske C.G., Harbarth S., Hindler J.F., Kahlmeter G., Olsson-Liljequist B. (2012). Multidrug-Resistant, Extensively Drug-Resistant and Pandrug-Resistant Bacteria: An International Expert Proposal for Interim Standard Definitions for Acquired Resistance. Clin. Microbiol. Infect..

[48] Adekanmbi A.O., Falodun O.I. (2015). Heavy Metals and Antibiotics Susceptibility Profiles of *Staphylococcus aureus* Isolated from Several Points Receiving Daily Input from the Bodija Abattoir in Ibadan, Oyo State, Nigeria. Adv. Microbiol..

[49] Dweba C.C., Zishiri O.T., el Zowalaty M.E. (2019). Isolation and Molecular Identification of Virulence, Antimicrobial and Heavy Metal Resistance Genes in Livestock-Associated Methicillin-Resistant *Staphylococcus aureus*. Pathogens.

[50] Igbinosa E.O., Beshiru A. (2019). Antimicrobial Resistance, Virulence Determinants, and Biofilm Formation of Enterococcus Species from Ready-to-Eat Seafood. Front. Microbiol..

[51] Ramnath L., Sithole B., Govinden R. (2017). Identification of Lipolytic Enzymes Isolated from Bacteria Indigenous to Eucalyptus Wood Species for Application in the Pulping Industry. Biotechnol. Rep..

[52] Al-Dhabi N.A., Esmail G.A., Ghilan A.K.M., Arasu M.V., Duraipandiyan V., Ponmurugan K. (2020). Isolation and Purification of Starch Hydrolysing Amylase from Streptomyces Sp. Al-Dhabi-46 Obtained from the Jazan Region of Saudi Arabia with Industrial Applications. J. King Saud. Univ. Sci..

[53] Lee J.S., Bae Y.M., Han A., Lee S.Y. (2016). Development of Congo Red Broth Method for the Detection of Biofilm-Forming or Slime-Producing *Staphylococcus* sp. LWT.

[54] Sakr A., Brégeon F., Mège J.L., Rolain J.M., Blin O. (2018). *Staphylococcus aureus* Nasal Colonization: An Update on Mechanisms, Epidemiology, Risk Factors, and Subsequent Infections. Front. Microbiol..

[55] van den Eede A., Hermans K., van den Abeele A., Floré K., Dewulf J., Vanderhaeghen W., Crombé F., Butaye P., Gasthuys F., Haesebrouck F. (2012). Methicillin-Resistant *Staphylococcus aureus* (MRSA) on the Skin of Long-Term Hospitalised Horses. Vet. J..

[56] Burton S., Reid-Smith R., McClure J.T., Weese J.S. (2008). *Staphylococcus aureus* Colonization in Healthy Horses in Atlantic Canada. Can. Vet. J..

[57] Claus F., Sachse A., Ried W. (2014). On the Economic Burden of MRSA in Germany. Gesundheitswesen.

[58] Weese J.S., Rousseau J., Willey B.M., Archambault M., McGeer A., Low D.E. (2006). Methicillin-Resistant *Staphylococcus aureus* in Horses at a Veterinary Teaching Hospital: Frequency, Characterization, and Association with Clinical Disease. J. Vet. Intern. Med..

[59] Soimala T., Lübke-Becker A., Schwarz S., Feßler A.T., Huber C., Semmler T., Merle R., Gehlen H., Eule J.C., Walther B. (2018). Occurrence and Molecular Composition of Methicillin-Resistant *Staphylococcus aureus* Isolated from Ocular Surfaces of Horses Presented with Ophthalmologic Disease. Vet. Microbiol..

[60] Baptiste K.E., Williams K., Willams N.J., Wattret A., Clegg P.D., Dawson S., Corkill J.E., O’neill T., Hart C.A. (2005). Methicillin-Resistant Staphylococci in Companion Animals. Emerg. Infect. Dis..

[61] Maddox T.W., Clegg P.D., Diggle P.J., Wedley A.L., Dawson S., Pinchbeck G.L., Williams N.J. (2012). Cross-Sectional Study of Antimicrobial-Resistant Bacteria in Horses. Part 1: Prevalence of Antimicrobial-Resistant Escherichia Coli and Methicillin-Resistant *Staphylococcus aureus*. Equine Vet. J..

[62] Momoh A.H., Kwaga J.K.P., Bello M., Sackey A.K.B., Larsen A.R. (2018). Antibiotic Resistance and Molecular Characteristics of *Staphylococcus aureus* Isolated from Backyard-Raised Pigs and Pig Workers. Trop. Anim. Health Prod..

[63] Lee A.S., de Lencastre H., Garau J., Kluytmans J., Malhotra-Kumar S., Peschel A., Harbarth S. (2018). Methicillin-Resistant *Staphylococcus aureus*. Nat. Rev. Dis. Prim..

[64] Scholtzek A.D., Hanke D., Walther B., Eichhorn I., Stöckle S.D., Klein K.S., Gehlen H., Lübke-Becker A., Schwarz S., Feßler A.T. (2019). Molecular Characterization of Equine *Staphylococcus aureus* Isolates Exhibiting Reduced Oxacillin Susceptibility. Toxins.

[65] Guérin F., Fines-Guyon M., Meignen P., Delente G., Fondrinier C., Bourdon N., Cattoir V., Léon A. (2017). Nationwide Molecular Epidemiology of Methicillin-Resistant *Staphylococcus aureus* Responsible for Horse Infections in France. BMC Microbiol..

[66] Scott H.M., Acuff G., Bergeron G., Bourassa M.W., Gill J., Graham D.W., Kahn L.H., Morley P.S., Salois M.J., Simjee S. (2019). Critically Important Antibiotics: Criteria and Approaches for Measuring and Reducing Their Use in Food Animal Agriculture. Ann. N. Y. Acad. Sci..

[67] World Health Organization (2019). WHO List of Critically Important Antimicrobials for Human Medicine (WHO CIA List).

[68] Bordin A.I., Huber L., Sanz M.G., Cohen N.D. (2022). Rhodococcus Equi Foal Pneumonia: Update on Epidemiology, Immunity, Treatment and Prevention. Equine Vet. J..

[69] Nehal M.F., Kamelia M.O., Azza N.F., Shaimaa R.A.A.E., el Shafii Soumaya S.A., Shahein M.A., Ibraheem E.M. (2021). Phenotypic Study on the Bacterial Isolates from Equine with Respiratory Disorders Regarding Antimicrobial Drug Resistance. World’s Vet. J..

[70] Weese J.S., Archambault M., Willey B.M., Dick H., Hearn P., Kreiswirth B.N., Said-Salim B., McGeer A., Likhoshvay Y., Prescott J.F. (2005). Methicillin-Resistant *Staphylococcus aureus* in Horses and Horse Personnel, 2000–2002. Emerg. Infect. Dis..

[71] Xue H., Wu Z., Li L., Li F., Wang Y., Zhao X. (2015). Coexistence of Heavy Metal and Antibiotic Resistance within a Novel Composite Staphylococcal Cassette Chromosome in a Staphylococcus Haemolyticus Isolate from Bovine Mastitis Milk. Antimicrob. Agents Chemother..

[72] Edogbo B., Okolocha E., Maikai B., Aluwong T., Uchendu C. (2020). Risk Analysis of Heavy Metal Contamination in Soil, Vegetables and Fish around Challawa Area in Kano State, Nigeria. Sci. Afr..

[73] Anyanwu B.O., Ezejiofor A.N., Igweze Z.N., Orisakwe O.E. (2018). Heavy Metal Mixture Exposure and Effects in Developing Nations: An Update. Toxics.

[74] Idrees M., Sawant S., Karodia N., Rahman A. (2021). *Staphylococcus aureus* Biofilm: Morphology, Genetics, Pathogenesis and Treatment Strategies. Int. J. Environ. Res. Public Health.

[75] Płusa T. (2019). The Importance of Biofilm in the Context of Increasing Bacterial Resistance to Antibiotics. Pol. Merkur. Lekarski.

[76] Pietrocola G., Nobile G., Rindi S., Speziale P. (2017). *Staphylococcus aureus* Manipulates Innate Immunity through Own and Host-Expressed Proteases. Front. Cell. Infect. Microbiol..

[77] Cotar A.I., Chifiriuc M.C., Dinu S., Bucur M., Iordache C., Banu O., Dracea O., Larion C., Lazar V. (2010). Screening of Molecular Virulence Markers in *Staphylococcus aureus* and Pseudomonas Aeruginosa Strains Isolated from Clinical Infections. Int. J. Mol. Sci..

